# Construction of a new T7 promoter compatible *Escherichia coli* Nissle 1917 strain for recombinant production of heme-dependent proteins

**DOI:** 10.1186/s12934-020-01447-5

**Published:** 2020-10-06

**Authors:** Kerstin Fiege, Nicole Frankenberg-Dinkel

**Affiliations:** Fachbereich Biologie, Abt. Mikrobiologie, Technische Universität Kaiserslautern, Paul-Ehrlich-Str. 23, 67663 Kaiserslautern, Germany

**Keywords:** *Escherichia coli* nissle 1917, T7 promoter, EcN(T7), Heme protein, Gene expression, Recombinant protein production

## Abstract

**Background:**

Heme proteins and heme-derived molecules are essential in numerous cellular processes. Research into their in vitro functionality requires the production of large amounts of protein. Unfortunately, high yield expression is hampered by the lack of *E. coli* strains naturally capable of taking up heme from the medium. We recently reported the use of the probiotic *E. coli* strain Nissle 1917 (EcN) to sufficiently produce heme containing proteins, as it encodes the outer membrane heme receptor, ChuA, which allows for natural uptake of heme. The EcN strain however lacks the gene for T7 RNA polymerase, which is necessary for the expression of genes under the control of the T7-promotor, widely used in expression vectors like the pET or pDuet series.

**Results:**

A new T7-promoter compatible EcN strain was constructed by integrating the gene for T7-RNA polymerase under the control of a *lac*UV5 promoter into the *malEFG* operon of EcN. Test expressions of genes via T7 promoter-based vectors in the new EcN(T7) strain were successful. Expression in EcN(T7) resulted in the efficient production of recombinant heme proteins in which the heme cofactor was incorporated during protein production. In addition, the new EcN(T7) strain can be used to co-express genes for the production of heme-derived molecules like biliverdin or other linear tetrapyrroles. We demonstrate the successful recombinant production of the phytochromes BphP, from *Pseudomonas aeruginosa,* and Cph1, from *Synechocystis* sp. PCC6803, loaded with their linear tetrapyrrole cofactors, biliverdin and phycocyanobilin, respectively.

**Conclusion:**

We present a new *E. coli* strain for efficient production of heme proteins and heme-derived molecules using T7-promoter based expression vectors. The new EcN(T7) strain enables the use of a broader spectrum of expression vectors, as well as the co-expression of genes using the pDuet expression vectors, for expressing heme containing proteins. By utilizing *E. coli* strains EcN and EcN(T7), capable of being fed heme, the rate limiting step of heme biosynthesis in *E. coli* is eliminated, thereby permitting higher heme saturation of heme proteins and also higher yields of heme-derived molecules.

## Background

Heme and heme degradation products play important roles in several biological processes. Firstly, as a cyclic tetrapyrrole cofactor in heme proteins, it is involved in processes, such as electron transfer and cell respiration (cytochromes), oxygen binding and transport (hemoglobin, myoglobin), production and sensing of nitric oxide (NO synthase, heme/nitric oxide/oxygen (H-NOX) proteins) or signal transduction (CooA, FixL) [1–5]. Secondly, heme is a precursor molecule for the formation of linear tetrapyrroles which are used as light-harvesting pigments in cyanobacteria and algae [1–6] or light-sensing chromophores in phytochrome-like photoreceptors in plants, algae, bacteria and fungi [7, 8].

In order to analyze the function of all these proteins and heme-derived molecules, recombinant protein production in *E. coli* is a widely used method [9]. Many different strains have been established in the past to obtain properly folded and active proteins, however, the production of active and cofactor loaded proteins is often limited to the availability of heme inside the *E. coli* cells [10]. This limitation is frequently bypassed through the addition of the heme cofactor to the cell-free lysate or purified protein. Drawback of this approach is a possible abnormal incorporation of the cofactor into the protein. Several methods have been developed in the past to obtain correctly folded and active proteins loaded with heme cofactor [11, 12], however many still do not yield 100% reconstitution with the cofactor. Furthermore, the formation of heme-derived molecules in *E. coli*, such as phycobilins, is dependent on heme biosynthesis of *E. coli* cells which is often rate limiting [13]. For the production of higher yields of these molecules, an increased heme availability inside the cell would be an advantage.

We have previously shown that the *E. coli* strain Nissle 1917 (EcN) is able to take up heme from the growth medium, thereby increasing the heme concentration inside the cell. This approach permits the successful saturation of expressed proteins with the heme cofactor during protein production. In this way, the cofactor is incorporated in its natural conformation during protein synthesis than if added to the completely folded protein afterwards [14]. Unfortunately, the EcN strain lacks the gene for T7 RNA polymerase, essential for the use of T7 promoter-based expression vectors such as the widely used pET system or pDuet-vectors [15]. In order to overcome this limitation, we integrated the T7 RNA polymerase gene, under control of the inducible *lac*UV5 promoter, into the genome of EcN. Thereby an EcN(T7) strain was constructed which broadens the spectrum of possibilities to produce heme proteins as well as heme derived molecules in vivo.

## Materials and methods

### Used strains and expression plasmids

*Escherichia coli* Nissle 1917 (EcN) [16] served as the parental strain for the construction of the new EcN(T7) strain. Test expressions were performed using *E. coli* BL21(DE3), EcN and EcN(T7) (Additional file 1: Table S1). All plasmids used (Additional file 1: Table S2) were verified via sequencing (GATC Eurofins genomics, Cologne; Seq-IT, Kaiserslautern).

### Construction of the expression cassette

Genomic DNA from *E. coli* BL21(DE3) was used as a template to obtain the T7-RNA polymerase gene. All oligonucleotides used in the construction are listed in Additional file 1: Table S3. Via two PCR reactions overlapping primers (*lac*-op-fwd, *lac*UV5-*Hind*III-fwd) were used to add the *lac* operator and *lac*UV5 promoter to the 5′-end of the T7 RNA polymerase gene to allow for isopropyl-β-d-thiogalactopyranoside (IPTG) inducible gene expression. A kanamycin resistance cassette, with flanking FRT sites, was amplified from pKD13 and ligated via a *Sal*I restriction site to the 3′-end of the T7-RNA polymerase gene in order to generate a selection marker for the following homologous recombination reaction. The thus obtained *lac*UV5-T7-FRT-*kan*-FRT (T7/Kan) fragment was purified via gel extraction and blunt end cloned into the plasmid pYP168 [17] via a *Sma*I restriction site. This product (pUC-T7-FRT-*kan*) was then used as a PCR template to add 50 bp of homologous sequences from the *malEFG* operon of *E. coli* Nissle 1917 (T7-*mal*-fwd and T7-*mal*-rev) to the expression cassette at both ends. The fragment was purified via agarose gel extraction.

### Chromosomal insertion of T7-RNA polymerase via homologous recombination

**Fig. 1:**
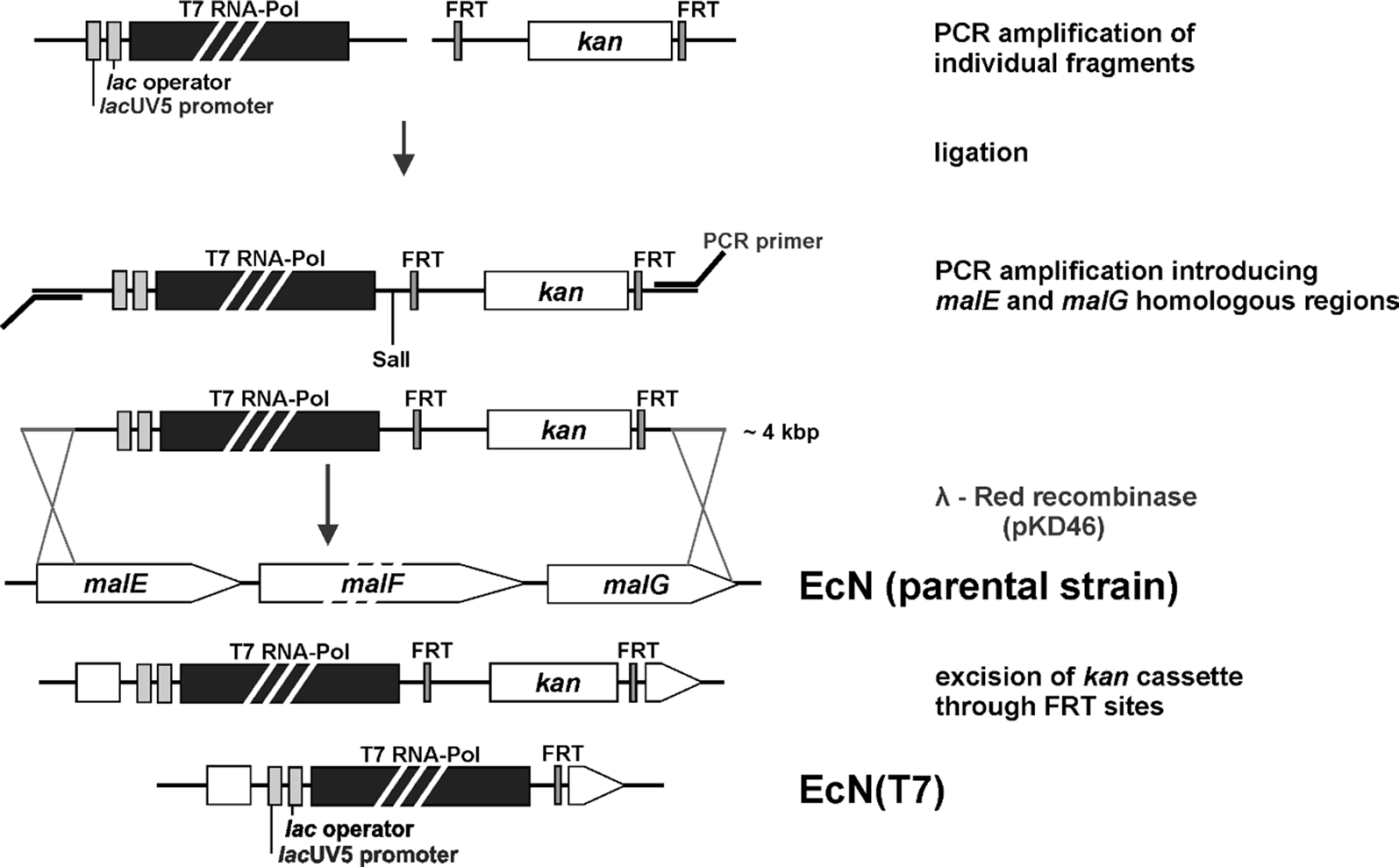
Construction scheme of the T7 promoter compatible EcN strain. A *lac*UV5 promoter was added to the 5′ end of the T7 RNA polymerase gene and ligated to a kanamycin resistance cassette flanked by FRT recognition sites. The ligated fragment was amplified via PCR and sequences homologous to the *malEFG* operon were added to both ends. The fragment containing the T7 RNA polymerase gene, as well as the FRT flanked resistance cassette, was introduced into the chromosome of EcN via homologous recombination using the λ-Red recombinase system. The kanamycin resistance cassette was removed via the FRT recognition sites resulting in the new strain EcN(T7)

For the insertion of the T7/Kan expression cassette into EcN, the λ-Red recombinase system was used as described previously (Fig. 1) [18, 19]. The insertion cassette was introduced via site-specific homologous recombination into the *malEFG* operon of *E. coli* Nissle 1917 (oligonucleotides T7-*mal*-fwd and T7-*mal*-rev). Insertion of the resistance cassette and loss of *malEFG* operon was verified by plating transformation reactions onto MacConkey agar plates containing 1% maltose and 50 µg/ml kanamycin. The kanamycin cassette was removed via flanked FRT recombination sites to obtain a markerless mutant using pCP20 [20]. The correct insertion of the T7 RNA polymerase gene was verified via sequencing (Eurofins Genomics).

### Test production of T7-RNA-polymerase induced protein production

T7 based expression vectors from our lab collection (see Additional file 1: Table S2) were transformed into BL21(DE3) as a positive control, EcN as a negative control and into the newly constructed EcN(T7) strain. Test productions were performed in 50 ml Luria–Bertani (LB) broth containing the appropriate antibiotics and 100 mM sorbitol. For Rdms_O216K LB high salt medium (0.5 M NaCl) without sorbitol was used. Cultures were incubated at 37 °C up to an OD_600_ of 0.7 for BL21(DE3) and OD_600_ of 1.2 for EcN and EcN(T7). Before induction, cultures were cooled down to 17 °C. Expression was induced by adding 0.5 mM IPTG for pACYC-*rdmS*_O216K and 0.1 mM IPTG for pTD-*ho1*, pET-cph1 and pACYC-*ho1-pcyA* to the culture. Expression of *bphP* was induced with 200 ng/ml anhydrotetracycline. For the production of phycocyanobilin (PCB) and biliverdin (BV) for the holo-phytochrome increasing amounts of hemin (in DMSO) were added in 2 h after induction. Cultures were incubated at 17 °C, 160 rpm overnight, harvested for 10 min, 9000 rpm (Sorvall LYNX 6000, F14 rotor), 4 °C and stored at -20 °C. Samples were taken before induction and after overnight incubation and were diluted to an OD_600_ of 0.5. Cell pellets were disrupted by sonification and separated via SDS-PAGE. Subsequently, the separated proteins were either stained with Coomassie Brilliant Blue or detected using a Western blot with the appropriate antibodies.

### Production and purification of recombinant produced *StrepII*-tagged proteins

For purification of RdmS_O216K, production was conducted in 2 L LB high salt medium. Cells were incubated as described before [21]. Immediately after induction, 10 µM hemin was added for the production of the holo-protein. Cell pellets were suspended in buffer W (100 mM Tris/HCl, pH 8.0, 150 mM NaCl, 1 mM EDTA, 10% glycerol) and 1 mM DTT, 0.25 mM 4-(2-aminoethyl) benzene-sulfonyl fluoride and spatula tip of DNase I and lysozyme were added. Cells were incubated on ice for 30 min and disrupted via a Microfluidizer LM20 (Microfluidics Corp., Westwood, MA, USA) at 15,000 PSI. Cell debris were removed via centrifugation at 4 °C and 19,000 rpm for 1 h (Sorvall LYNX 6000, T29 rotor). A Strep-Tactin chromatography column (IBA GmbH, Göttingen), equilibrated with buffer W, was used for affinity chromatography. Unwanted proteins were removed by washing with 10 column volumes of buffer W. Elution of *StrepII*-tagged proteins was performed with buffer E (buffer W containing 2.5 mM D-desthiobiotin). Elution fractions containing the desired protein were dialyzed against 20 mM TES buffer, pH 8.0, containing 100 mM KCl and 10% glycerol. Proteins were concentrated using Amicon concentrator devices (molecular weight cut-off 100,000; Merck).

### UV–visible spectroscopy

UV–vis spectroscopy was performed using an 8453 UV–visible spectrophotometer (Agilent Technologies). Heme spectra were taken at room temperature in 20 mM TES buffer pH 8.0 containing 100 mM KCl and 10% glycerol. Spectra were taken from 350–700 nm under oxidizing conditions. Phytochrome spectra were taken at 25 °C using cell-free lysates (100 mM Tris/HCl pH 8.0, 150 mM NaCl, 1 mM EDTA). The BphP samples were incubated for 3 min with red light at 690 nm for Pfr spectra and 3 min with far-red light at 750 nm for Pr spectra as described previously [22]. The Pfr spectra were subtracted from the Pr spectra to obtain the red/far-red induced difference spectra. For Cph1, difference spectra were obtained in a similar way, expect that the red and far-red light filters of 630 and 730 nm were used, respectively [23]. To analyze the saturation of Cph1 with its chromophore, difference spectra were measured again after 30 min incubation with 40 µM phycocyanobilin (PCB) for 30 min at room temperature.

## Results and discussion

### Construction of the new EcN(T7) strain

We have recently shown that *E. coli* Nissle 1917 (EcN) is a very suitable host for the production of heme proteins [14]. In this study, we went a step further and constructed a T7 promoter compatible strain to overcome the limitation of recombinant protein production in EcN to expression vectors lacking the T7 promoter. To this end, the method of Albermann et al. was chosen [18] to obtain a stable chromosomal integration of a PCR fragment into EcN. This method uses non-essential sugar degradation genes for site-specific integration of recombinant genes. By monitoring the ability of sugar degradation, the gene integration can be detected on sugar containing indicator plates. Therefore, the *malEFG* operon of EcN was chosen as the site of integration. For an IPTG inducible expression of the T7 RNA polymerase, the gene was set under the control of a *lac*UV5 promoter and ligated to a kanamycin resistance cassette flanked by FRT recombination sites (Fig. 1). Homologous sequences to the *malEFG* operon of EcN were added at both sites for a double homologous recombination event. Integration of the PCR fragment was performed using the λ-Red recombinase system [19]. Integration of the T7/Kan fragment, therefore, led to a deletion of the *malEFG* operon. The kanamycin cassette was removed via the FRT sites using the FRT/Flp-recombination system [20] resulting in anew EcN(T7) Δ*malEFG* strain (Fig. 1). After removing the kanamycin cassette and verification by sequencing the successful construction of the EcN(T7) was tested.

### T7-promoter dependent production of a heme protein

**Fig. 2:**
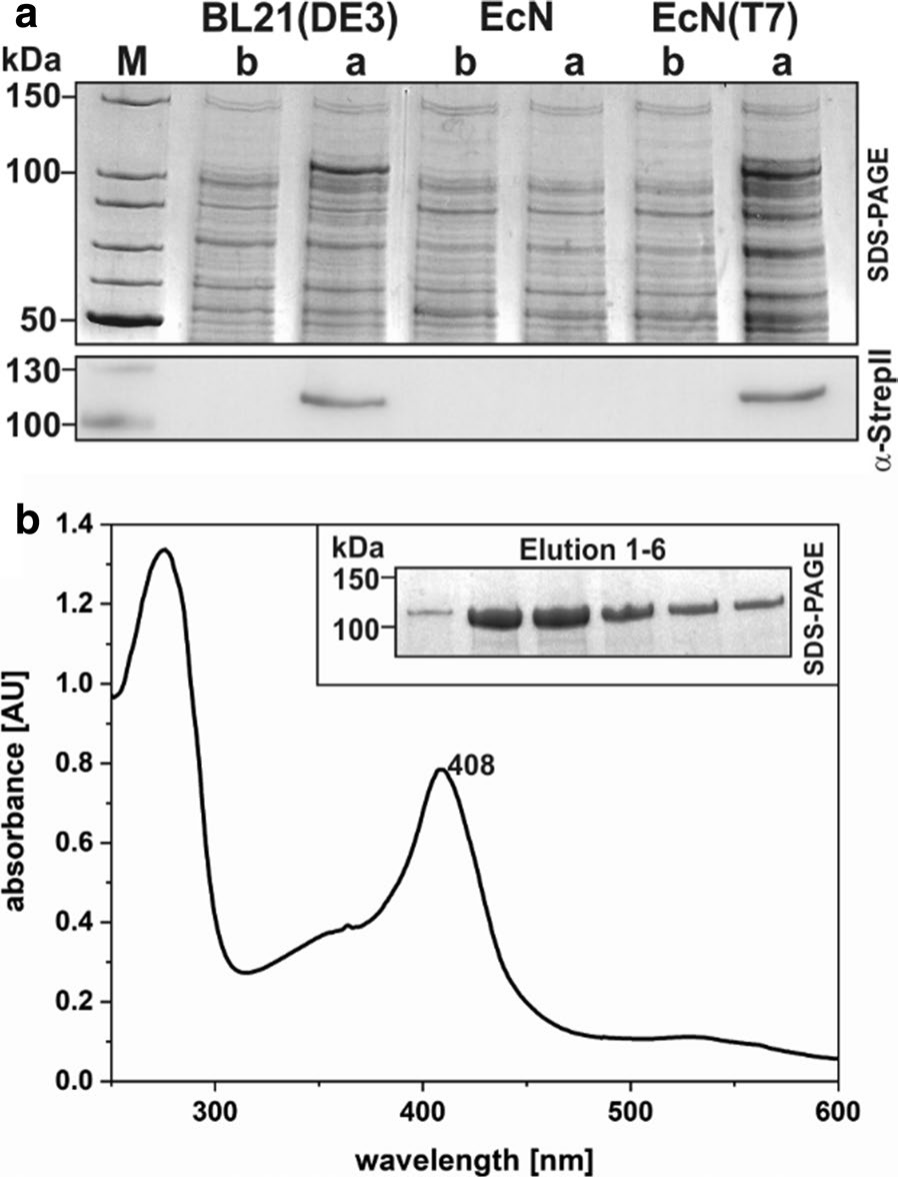
Test expression of *rdmS* (T7 promoter) and UV/vis heme spectrum. **a** SDS-PAGE and Western blot targeting the C-terminal StrepII-tag of *rdmS* expressed in the *E. coli* strains BL21(DE3), EcN and the newly constructed EcN(T7). Samples were taken before (b) and after (a) induction with 0.5 mM IPTG. Test expressions were performed at 17 °C overnight. **b** UV/vis spectrum of 20 µM RdmS produced in EcN(T7) with addition of 10 µM hemin to the growth medium. Shown are the peaks for proteins at 280 nm and the heme Soret band at 408 nm. The inset shows an SDS-PAGE of the elution fractions of the affinity purified RdmS. Known molecular weights of standard proteins are marked

As EcN was shown to be an efficient strain for recombinant production of heme proteins, first test expressions with EcN(T7) were performed with the sensor kinase RdmS from *M. acetivorans* [21], that contains a covalently bound heme cofactor. Experiments were performed with the O216K variant of RdmS as previously described [21]. Test expressions of *rdmS-*StrepII via the T7-promoter based vector pACYCduet1 resulted in detectable levels of expression and protein production in the commonly used *E. coli* strain BL21(DE3), which served as a positive control. In addition, expression and subsequent protein production was also observed in the newly constructed EcN(T7) strain. In contrast, the parental EcN strain was unsuitable for expressing the same construct as it lacks the T7 RNA polymerase (Fig. 2a). Production of RdmS-StrepII in EcN(T7), with concomitant addition of heme to the growth medium, led to the efficient incorporation of the heme cofactor into the protein during production. UV/vis spectroscopy of purified RdmS-StrepII showed a typical heme spectrum for RdmS, with a Soret band at 408 nm displaying the Fe(III) state of the heme cofactor in RdmS (Fig. 2b). The new EcN(T7) strain can therefore be used for the efficient production of heme proteins without heme reconstitution after protein production.

### EcN(T7), a new strain for the production of linear tetrapyrroles

**Fig. 3:**
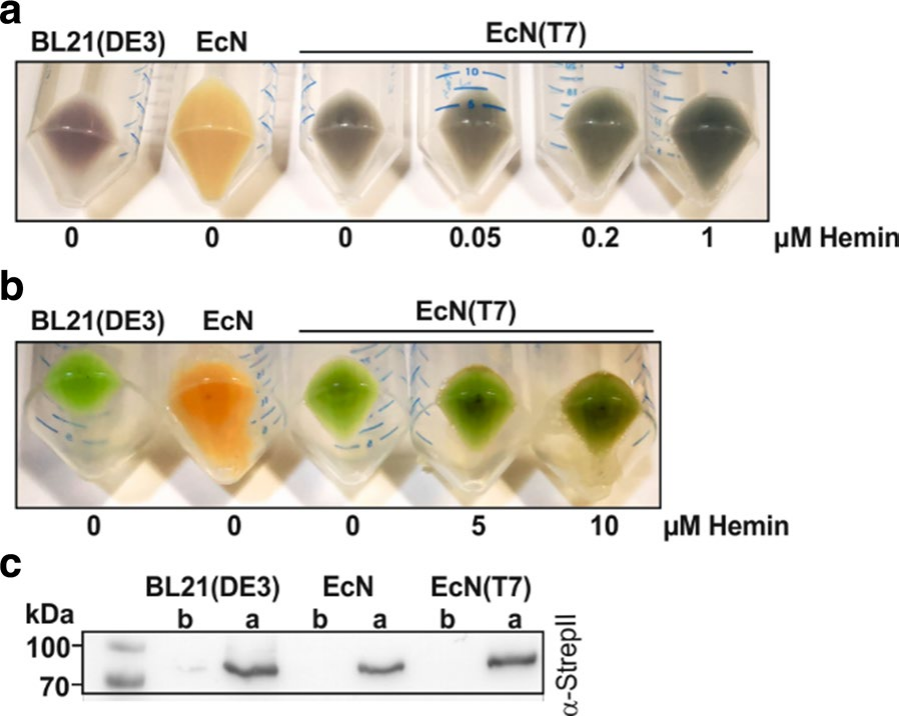
Test expressions of the genes encoding for heme oxygenase HO1 and phycocyanobilin:ferredoxin oxidoreductase PcyA. Expressions were performed with three *E. coli* strains: BL21(DE3), EcN and the newly constructed EcN(T7) strain. Different concentrations of hemin were added to the EcN(T7) cultures. T7-based expression was induced by 0.1 mM IPTG. **a** Test expression of *ho1* and *pcyA* for phycocyanobilin (PCB) production (T7 promoter). **b** Test expressions of *ho1* (T7 promoter) for biliverdin production and co-production of holo-phytochrome BphP (tet promoter) from *Pseudomonas aeruginosa.* **c** Verification of BphP (~ 81 kDa) production via StrepII-tag (tet promoter). Samples were taken before (b) and after (a) induction with anhydrotetracycline at 17 °C overnight. Known molecular weights of standard proteins are marked

We have previously demonstrated the large-scale production of the linear tetrapyrrole molecule, phycoerythrobilin, using a pDuet based expression system and *E. coli* BL21(DE3) [24]. In a similar way, we tested the use of the newly constructed EcN(T7) for the production of the linear tetrapyrrole molecule, phycocyanobilin (PCB). To do so, the genes encoding heme oxygenase, HO1, and the phycocyanobilin:ferredoxin oxidoreductase, PcyA, from cyanophages (pACYC*-ho1-pcyA*) was used to test the formation of biliverdin IXα (BV) and the following conversion to PCB [25]. Whereas test expression in the parental EcN strain resulted in the typical beige-colored cell pellet, the EcN(T7) and BL21(DE3) cell pellets exhibited a bluish color indicating the successful synthesis of PCB in both strains (Fig. 3a). Furthermore, addition of increasing amounts of hemin to the cultures appeared to result in slightly darker blue cell pellets.

**Fig. 4:**
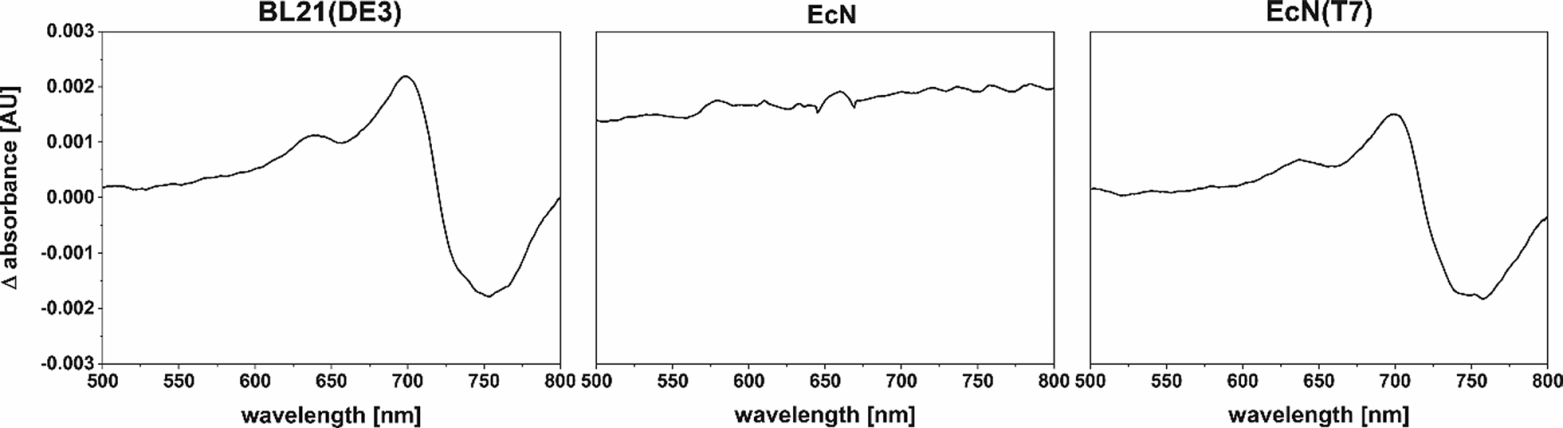
Red-Farred induced difference spectra of the phytochrome BphP from *P. aeruginosa*. BphP was co-produced with the heme oxygenase HO1 (T7 promoter) for biliverdin production in *E. coli* BL21(DE3), EcN and EcN(T7). The calculated difference spectra were smoothed using a 25 points Savitzky-Golay filter

In a further approach, the new EcN(T7) strain was used to coexpress genes on two vectors using different inducible promoters. Coexpression of the genes for HO1 (T7 promoter) and the phytochrome BphP (tet-promoter) from *Pseudomonas aeruginosa* resulted in greenish colored cell pellets, indicating the formation of BV (Fig. 3b). As already shown for the formation of PCB, increasing the amount of added hemin to the cell culture resulted in a stronger coloring of the cell pellets. This observation led to the assumption that the ability of EcN(T7) (and the parental EcN strain) to take up heme from the growth medium, resulted in a higher hemin supply for the conversion into linear tetrapyrroles. Especially for the production of heme-derived molecules in batch cultures, EcN(T7) might be an improved strain to obtain higher yields of product. However, coexpression of *ho1* and *bphP* did not only lead to the formation of BV, but also to the formation of holo-BphP. As *bphP* expression was induced via a tet-promoter, the protein was not only detectable in the T7 promoter dependent *E. coli* strains, but also in the parental EcN strain via a fused StrepII-tag (Fig. 3c). Binding of BV as the red-light detecting chromophore of BphP, was confirmed by difference spectra of the Pr and Pfr states of BV [22]. Typical difference spectra of holo-phytochrome were observed for the positive control BL21(DE3) and the newly constructed EcN(T7) strain, whereas for the original EcN strain, no difference spectrum was detectable (Fig. 4).

**Fig. 5:**
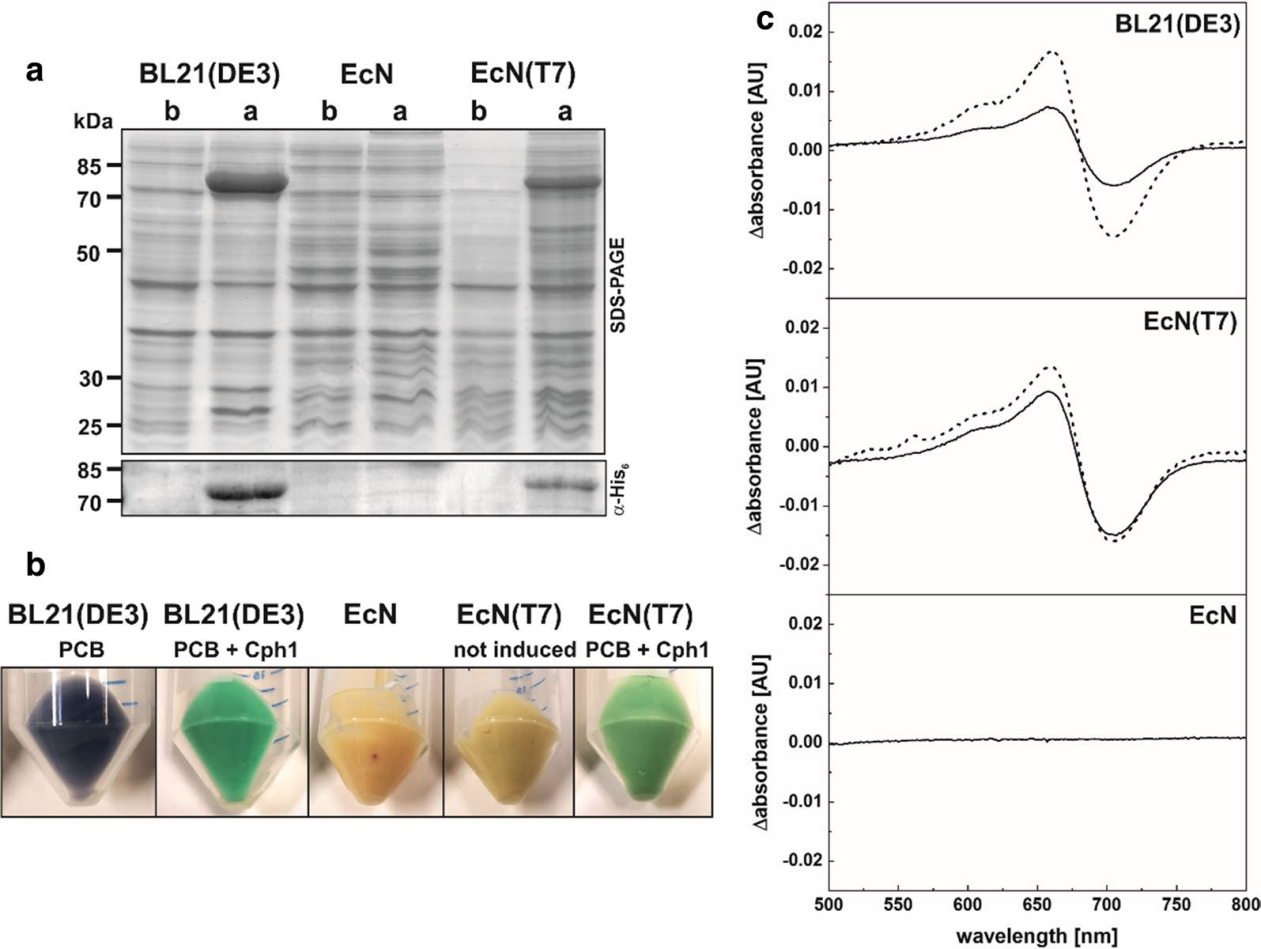
Test expressions of the genes encoding for heme oxygenase, HO1, phycocyanobilin:ferredoxin oxidoreductase, PcyA, and cyanobacterial phytochrome, Cph1 (all under control of the T7 promoter). **a** Test expressions were performed with three *E. coli* strains: BL21(DE3), EcN and the newly constructed EcN(T7) strain. Verification of Cph1 (~ 80 kDa) production via His_6_-tag. Samples were taken before (b) and after (a) induction with 0.5 mM IPTG at 17 °C overnight. Known molecular weights of standard proteins are marked. **b** Colored pellets of test expressions of different combinations of PCB and holo-Cph1 in different *E. coli* strains. **c** Difference spectra of holo-Cph1 produced in different strains. Spectra were taken from cell-free lysate and adjusted to the total protein concentration: holo-Cph1 before (solid line) and after incubation with additional PCB (dashed line)

In a similar way, we also tested the expression of holo-cyanobacterial phytochrome Cph1 [26]. Again, the use of EcN(T7) proved to be useful in the expression of holo-Cph1 through the coexpression of genes for chromophore biosynthesis (*ho1* and *pcyA*; see also Fig. 3) and apo-*cph*1. Coexpression of *ho1*, *pcyA* and *cph1* resulted in typical colored cells for holo-Cph1. Comparison of difference spectra before and after incubation with PCB indicated a higher saturation of Cph1 after production in EcN(T7) than in BL21(DE3) (Fig. 5).

## Conclusion

Here we present a novel tool to produce recombinant heme dependent proteins in *E. coli* strain Nissle 1917 (EcN), as it naturally takes up heme from the surrounding medium. Our approach of integrating the T7-RNA polymerase gene under the control of an IPTG inducible promoter, into EcN permits the use of T7 promoter-based expression systems. Additionally, the strain might be well suited for high yield production of heme-derived molecules in batch cultures through feeding with external heme as it grows to high cell densities compared to BL21(DE3). In summary, the EcN(T7) strain broadens the spectrum of expression strains available to produce recombinant heme proteins and other heme-derived molecules.

## Supplementary information

**Additional file 1: Table S1.** *E. coli* strains used in this study. **Table S2.** Plasmids used in this study. **Table S3.** Oligonucleotides used in this study.

## Data Availability

All material listed in the manuscript is available from the corresponding author.

## Abbreviations

a: After induction
b: Before induction
BV: Biliverdin IXα
EcN: *E. coli* Nissle 1917
IPTG: Isopropyl-β-d-thiogalactopyranoside
PCB: phycocyanobilin
Pfr: Far-red light absorbing phytrochrome form
Pr: Red light absorbing phytochrome form
