# Supplementary material for: Construction of a new T7 promoter compatible Escherichia coli Nissle 1917 strain for recombinant production of heme-dependent proteins
Source: Microb Cell Fact. 2020 Oct 6;19:190. doi: 10.1186/s12934-020-01447-5 (PMC7542351; doi:10.1186/s12934-020-01447-5)
Supplement: Supplementary file 1 — Additional file 1: Table S1. E. coli strains used in this study. Table S2. Plasmids used in this study. Table S3. Oligonucleotides used in this study. [file 12934_2020_1447_MOESM1_ESM.pdf]

Supplementary Information

Construction of a new T7 promoter compatible *Escherichia coli* Nissle 1917 strain for recombinant production of heme-dependent proteins

Kerstin Fiege and Nicole Frankenberg-Dinkel*

Technische Universität Kaiserslautern, Fachbereich Biologie, Abt. Mikrobiologie, Paul-Ehrlich-Str. 23, D-67663 Kaiserslautern, Germany

* Corresponding author: +49 631 2052353; nfranken@bio.uni-kl.de

Additional file 1: Table S1. *E. coli* strains used in this study

**strain**	**genotype**	**reference**
DH5α	F$^-$ *endA1 glnV44 thi-1 recA1 relA1 gyrA96 deoR nupG purB20* φ80d*lacZ*ΔM15 Δ(*lacZYA-argF*)U169, hsdR17($r_K^- m_K^+$), λ$^-$	[1]
BL21(DE3)	F$^-$ *ompT gal dcm lon hsdSB*(rB$^-$ mB$^-$) λ(DE3)	[2]
Nissle 1917 (EcN)	Serotype O6:K5:H1	[3]
EcN(T7)	EcN derivate, insertion of T7-RNA polymerase gene with *lac*UV5 promoter by deletion of *malEFG* operon	this study

Additional file 1: Table S2 Plasmids used in this study

**plasmid**	**relevant characteristics**	**reference**
pKD46	p_{araB} γ β exo (red recombinase), Amp^R	[4]
pKD13	template for Kan resistance cassette flanked by FRT sites	[4]
pCP20	FLP+, λ cl857$^+$, λ p_R Rep^{ts}, Amp^R, Km^R	[5]
pYP168	pUC-derivate, Vector for blunt end cloning via *Sma*I restriction site	[6]
pUC-T7-FRT-Kan	T7 RNA polymerase gene with *lac*UV5 promoter, Kan resistance cassette with flanking FRT sites, Amp^R	this study
pACYC-*rdmS*_O216K	C-terminal StrepII-tag, T7 promoter	Lab collection
pACYC-*ho1*-*pcyA*	pACYCduet1-based, cyanophage *ho1* from P-SSM2 and *pcyA* from P-SSM4	[7]
pTD-*ho1*	pACYCduet1-based, cyanophage *ho1* from P-SSM2	[7]
pASK-IBA3-*bphP*	C-terminal StrepII-tag, tet promoter; *P. aeruginosa bphP*	[8]
pET*cph*1	C-terminal His-tag, T7 promoter; *Synechocystis* sp. PCC6803 *cph*1	Lab collection

Additional file 1: Table S3. Oligonucleotides used in this study

**oligonucleotide**	**Sequence** (5'-3'), restriction site in italics	
*lac*UV5-*Hind*III-fwd	GCAT*AAGCTT*CCAGGCTTTACACTTTATGCTTCCGGCTCGTAT AATGTGTGGAATTGTGAGCGG	this study
lac-op-fwd	TATGTGTGGAATTGTGAGCGGATAACAATTTACTAACTGGAA GAGGCACTA	this study
T7-*SalI*-rev	GCTA*GTCGAC*TTACGCGAACGC	this study
Kan-*Sal*I-fwd	GGATCC*GTCGAC*TAGCCAGTTCGAAGTTCCTAT	this study
Kan-rev	GCTGCTTCGAAGTTCCTATAC	this study
T7-*mal*-fwd	AAGGTAAACTGGTAATCTGGATTAACGGCGATAAAGGCTATA ACGGTCTCGCTGCCAGGCTTTACACTTTATGCTTCCGGCTCG	this study
T7-*mal*-rev	GATCGGTAATGCAGACATCACGGCAGCGGCGGCAAAGTCAC CCCACAGGTAGTTTTACCGCATCAGGCGCTGCTTC	this study
mal-test-fwd	AGGATGGAAAGAGGTTGCCG	this study
mal-test-rev	GCAGCGATCCTGTTACTGGT	this study
T7-seq-rev	GTTGACGCTCAAACATCTTG	this study
T7-seq-fwd	ACCTTGCGTAGTTCCTCCTAAGC	this study
T7-seq2-fwd	ACCACGGCCTGAGCTATAACTG	this study
T7-seq3-fwd	CCG TGA CCT TGA AGC TAA GC	this study
T7-seq4-fwd	TGA TGG TTT CCC TGT GTG GC	this study

References

1. Grant SG, Jessee J, Bloom FR, Hanahan D: **Differential plasmid rescue from transgenic mouse DNAs into *Escherichia coli* methylation-restriction mutants.** *Proc Natl Acad Sci U S A* 1990, **87:**4645-4649.
2. Studier FW, Moffatt BA: **Use of bacteriophage T7 RNA polymerase to direct selective high-level expression of cloned genes.** *J Mol Biol* 1986, **189:**113-130.
3. Grozdanov L, Raasch C, Schulze J, Sonnenborn U, Gottschalk G, Hacker J, Dobrindt U: **Analysis of the genome structure of the nonpathogenic probiotic *Escherichia coli* strain Nissle 1917.** *J Bacteriol* 2004, **186:**5432-5441.
4. Datsenko KA, Wanner BL: **One-step inactivation of chromosomal genes in *Escherichia coli* K-12 using PCR products.** *Proc Natl Acad Sci U S A* 2000, **97:**6640-6645.
5. Cherepanov PP, Wackernagel W: **Gene disruption in *Escherichia coli*: TcR and KmR cassettes with the option of Flp-catalyzed excision of the antibiotic-resistance determinant.** *Gene* 1995, **158:**9-14.
6. Hoffmann MC, Wagner E, Langklotz S, Pfander Y, Hott S, Bandow JE, Masepohl B: **Proteome Profiling of the *Rhodobacter capsulatus* Molybdenum Response Reveals a Role of IscN in Nitrogen Fixation by Fe-Nitrogenase.** *J Bacteriol* 2015, **198:**633-643.
7. Dammeyer T, Bagby SC, Sullivan MB, Chisholm SW, Frankenberg-Dinkel N: **Efficient phage-mediated pigment biosynthesis in oceanic cyanobacteria.** *Curr Biol* 2008, **18:**442-448.
8. Tasler R, Moises T, Frankenberg-Dinkel N: **Biochemical and spectroscopic characterization of the bacterial phytochrome of *Pseudomonas aeruginosa*.** *FEBS J* 2005, **272:**1927-1936.
